# Association of Circulating Follistatin-Like 1 Levels with Inflammatory and Oxidative Stress Markers in Healthy Men

**DOI:** 10.1371/journal.pone.0153619

**Published:** 2016-05-04

**Authors:** Satoko Hayakawa, Koji Ohashi, Rei Shibata, Ryotaro Takahashi, Naoya Otaka, Hayato Ogawa, Masanori Ito, Noriyoshi Kanemura, Mizuho Hiramatsu-Ito, Nobuo Ikeda, Toyoaki Murohara, Noriyuki Ouchi

**Affiliations:** 1 Department of Cardiology, Nagoya University Graduate School of Medicine, Nagoya, Japan; 2 Molecular Cardiovascular Medicine, Nagoya University Graduate School of Medicine, Nagoya, Japan; 3 Department of Advanced Cardiovascular Therapeutics, Nagoya University Graduate School of Medicine, Nagoya, Japan; 4 Department of Cardiology, Chunichi Hospital, Nagoya, Japan; Osaka University Graduate School of Medicine, JAPAN

## Abstract

**Objectives:**

Follistatin-like 1 (Fstl1) is a circulating glycoprotein that plays a crucial role in cardiovascular diseases and inflammation-related disorders. We have shown that Fstl1 acts as an anti-inflammatory factor that protects against ischemic heart disease and chronic kidney disease. Here we examined whether plasma level of Fstl1 associates with markers of inflammation and oxidative stress in apparently healthy Japanese men.

**Methods and Results:**

Plasma Fstl1 levels were measured by enzyme-linked immunosorbent assay. Circulating Fstl1 concentrations positively correlated with levels of fasting immune-reactive insulin (FIRI), high-sensitive CRP (hsCRP) and derivatives of reactive oxidative metabolites (dROMs), an indicator of oxidative stress. The levels of hsCRP positively associated with Fstl1, body mass index (BMI), triglyceride, FIRI and dROMs levels. dROMs levels positively associated with Fstl1, Hemoglobin A1c and hsCRP levels. Multiple regression analysis with confounding factors revealed that Fstl1 levels, together with BMI and FIRI, correlated with hsCRP and that Fstl1 levels correlated with dROMs.

**Conclusion:**

Our observations indicate that measurement of plasma Fstl1 levels can be valuable for assessment of pro-inflammatory and oxidative stress conditions.

## Introduction

Chronic inflammation and oxidative stress are linked with a wide range of human diseases including cardiovascular disorders, metabolic dysfunction and chronic kidney disease [1–3]. Inflammatory process can induce oxidative stress and vice versa. These mutual accelerating actions may contribute to various pathological conditions.

Follistatin-like 1 (Fstl1), also known as TSC-36, is a secreted glycoprotein, which belongs to follistatin family [4]. Fstl1 is synthesized and secreted by various tissues including heart and skeletal muscle tissues. We have demonstrated that Fstl1 promotes endothelial cell survival and ischemia-induced revascularization process [5]. We have also shown that muscle-derived Fstl1 prevents neointimal formation in response to injury [6]. Furthermore, Fstl1 attenuates myocardial inflammation and injury following ischemia and prevents cardiac hypertrophy and dysfunction after pressure overload [7–10]. Recently, we have reported that cardiomyocyte-derived Fstl1 reduces renal inflammation and damage after subtotal nephrectomy [11]. Thus, Fstl1 can play an important role in cardiovascular protection. Clinically, circulating levels of Fstl1 are elevated in patients with acute coronary syndrome [12]. High levels of plasma Fstl1 are also observed in patients with chronic systolic heart failure [13]. These results indicate that Fstl1 may be a useful marker for evaluation of cardiovascular disease. A number of markers for inflammatory response and oxidative stress are associated with various chronic diseases including cardiovascular disorders and renal diseases [14–17]. Here, we investigated the association of circulating Fstl1 levels with markers of inflammation and oxidative stress in apparently healthy subjects.

## Materials and Methods

### Study population

In the present study, 87 male subjects, who visited Chunichi Hospital for a routine medical checkup, were enrolled between 2006 and 2009. All subjects had no history of cardiovascular disease and took no medication. All subjects enrolled in this study provided written informed consent. This study was approved by the ethics committee of Nagoya University School of Medicine and Chunichi Hospital.

### Measurement of clinical parameters

Blood samples were obtained from all subjects after an overnight fasting. Plasma Fstl1 levels were measured by enzyme-linked immunosorbent assay (ELISA) kit for human (USCN). The intra-assay and inter-assay coefficients of variation were less than 10% and 12%, respectively. Derivatives of reactive oxidative metabolites (dROMs) were determined by colorimetric assay, and expressed in Carratelli (Carr) units, where 1 Carr unit corresponds to 0.8 mg/l of hydrogen peroxide [18]. Standard assays were used to measure glucose, hemoglobin A1c (HbA1C), fasting immune-reactive insulin (FIRI), HDL cholesterol, LDL cholesterol, triglycerides, creatinine and high-sensitive C-reactive protein (hsCRP) levels. HbA1C levels were evaluated by Japan Diabetes Society values. Blood pressure (BP) was measured with an appropriate arm cuff and a mercury column sphygmomanometer after at least 10 minute rest in sitting position. Body mass index (BMI) was calculated as the ratio of weight (kg) to squared height (m^2^). Estimated glomerular filtration rates (eGFR) were evaluated by circulating creatinine (Cr) levels, age and sex according to the Simplified Modification of Diet in Renal Disease equation for Japanese. eGFR was calculated by 194 × Cr (mg/dl) ^-1.094^ × Age (years) ^-0.287^.

### Statistical analysis

All values were presented as mean ± standard deviation (SD) for continuous variables. Associations between Fstl1 and the parameters were examined by simple correlation analysis. Multiple regression analyses were performed to assess the correlations of the indicated parameters to plasma Fstl1 levels. Plasma levels of Fstl1, hsCRP, FIRI and triglyceride were logarithmically transformed (log_10_) because of their skewed distributions. A value of p <0.05 was considered as statistically significant. All analyses were performed using JMP pro (version 11; SAS Institute Inc).

## Results

**Fig 1:**
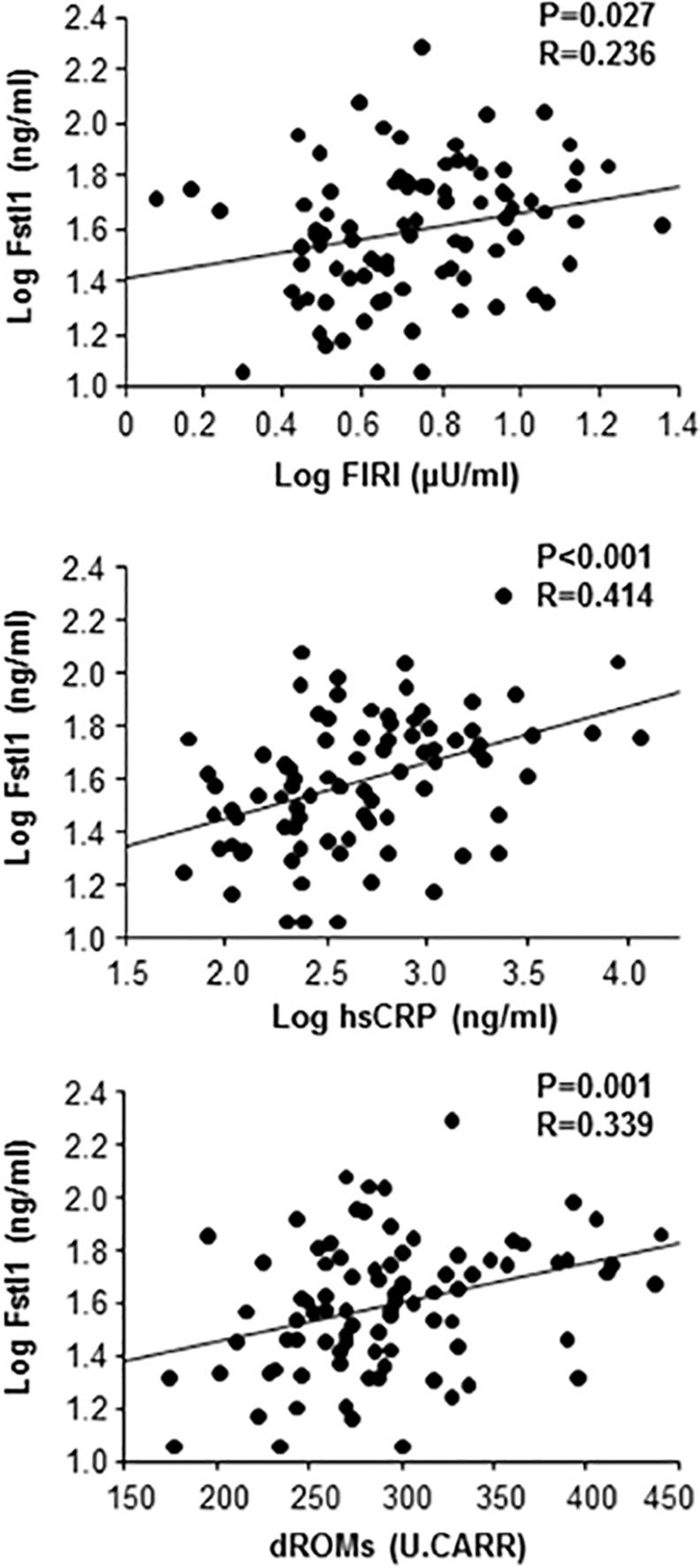
Correlation between plasma Fstl1 levels and clinical parameters. Correlation of plasma Log Fstl1 levels with Log fasting immune-reactive insulin (FIRI), Log high sensitive CRP (hsCRP) and derivatives of reactive oxidative metabolites (dROMs) was analyzed.

Clinical characteristics of all subjects are shown in Table 1. Mean values of BMI, systolic and diastolic BP, HbA1C, FBS, LDL cholesterol, HDL cholesterol, triglyceride, Cr and eGFR were within normal range (Table 1). Mean circulating Fstl1 level was 46.2±28.9 ng/ml. In single regression analysis for Fstl1, circulating Fstl1 levels were positively correlated with FIRI, hsCRP and dROMs (Table 2 and Fig 1). Multiple regression analysis with BMI, FIRI, hsCRP and dROMs showed that hsCRP and dROMs were significantly correlated with Fstl1 levels (Table 2).

**Table 1:** Clinical characteristics of all subjects.

Parameters	Subjects (n = 87)
Age (years)	52.7±8.9
BMI (kg/m^2^)	23.3±2.6
Systolic BP (mmHg)	114.9±15.8
Diastolic BP (mmHg)	71.8±10.7
Fasting glucose (mmol/l)	5.6±0.6
Hemoglobin A1c (%)	5.07±0.39
LDL cholesterol (mmol/l)	3.19±0.86
HDL cholesterol (mmol/l)	1.49±0.35
Log Triglyceride (mmol/l)	0.10±0.23
Creatinine (μmol/l)	75.8±12.1
eGFR (ml/min/1.73m^2^)	76.0±13.3
Log hsCRP (ng/ml)	2.66±0.49
Log FIRI (μU/ml)	0.73±0.25
dROMs (U.CARR)	292.7±56.9
Log Fstl1 (ng/ml)	1.59±0.25

BMI: body mass index, BP: blood pressure, LDL: low density lipoprotein, HDL: high density lipoprotein, eGFR: estimated glomerular filtration rate, hsCRP: high sensitive CRP, FIRI: fasting immune-reactive insulin, dROM: derivatives of reactive oxidative metabolites. Data are presented as means ± SD.

**Table 2:** Correlation with Log Fstl1 levels.

	Single		Multiple	
	r	P value	F	P value
Age	0.173	0.110		
BMI	0.181	0.093	0.000	0.993
Systolic BP	0.017	0.875		
Diastolic BP	0.057	0.603		
Fasting glucose	0.013	0.904		
Hemoglobin A1c	0.167	0.122		
LDL cholesterol	0.018	0.869		
HDL cholesterol	-0.040	0.716		
Log Triglyceride	0.038	0.724		
Creatinine	0.066	0.544		
eGFR	-0.122	0.262		
Log hsCRP	0.414	<0.001	11.125	0.001
Log FIRI	0.236	0.027	0.489	0.486
dROM	0.339	0.001	5.084	0.027

BMI: body mass index, BP: blood pressure, LDL: low density lipoprotein, HDL: high density lipoprotein, eGFR: estimated glomerular filtration rate, hsCRP: high sensitive CRP, FIRI: fasting immune-reactive insulin, dROM: derivatives of reactive oxidative metabolites. Multiple model includes all variables at baseline with p<0.1 by single analysis. Data are presented as means ± SD.

In single regression analysis for hsCRP, Fstl1, body mass index (BMI), triglyceride, FIRI and dROMs positively associated with hsCRP (Table 3). Multiple regression analysis with Fstl1, BMI, triglyceride, FIRI and dROMs showed that Fstl1, BMI and FIRI were significantly correlated with hsCRP. Single regression analysis for dROMs demonstrated that Fstl1, hemoglobin A1c and hsCRP positively associated with dROMs levels (Table 4). Multiple regression analysis with Fstl1, hemoglobin A1c and hsCRP showed that Fstl1 correlated with hsCRP.

**Table 3:** Correlation with Log hsCRP levels.

	Single		Multiple	
	r	P value	F	P value
Age	-0.046	0.671		
BMI	0.431	<0.001	5.608	0.020
Systolic BP	0.001	0.989		
Diastolic BP	-0.076	0.484		
Fasting glucose	-0.045	0.680		
Hemoglobin A1c	0.173	0.110		
LDL cholesterol	0.054	0.620		
HDL cholesterol	-0.152	0.160		
Log Triglyceride	0.265	0.013	1.747	0.190
Creatinine	-0.015	0.893		
eGFR	-0.002	0.985		
Log Fstl1	0.414	<0.001	7.350	0.008
Log FIRI	0.429	<0.001	4.359	0.040
dROM	0.322	0.002	3.248	0.075

BMI: body mass index, BP: blood pressure, LDL: low density lipoprotein, HDL: high density lipoprotein, eGFR: estimated glomerular filtration rate, hsCRP: high sensitive CRP, FIRI: fasting immune-reactive insulin, dROM: derivatives of reactive oxidative metabolites. Multiple model includes all variables at baseline with p<0.1 by single analysis. Data are presented as means ± SD.

**Table 4:** Correlation with dROMs levels.

	Single		Multiple	
	r	P value	F	P value
Age	0.015	0.892		
BMI	0.147	0.174		
Systolic BP	0.010	0.928		
Diastolic BP	0.023	0.835		
Fasting glucose	-0.108	0.320		
Hemoglobin A1c	0.243	0.023	2.849	0.095
LDL cholesterol	-0.041	0.703		
HDL cholesterol	-0.103	0.343		
Log Triglyceride	-0.029	0.794		
Creatinine	0.006	0.953		
eGFR	-0.030	0.784		
Log hsCRP	0.322	0.002	3.252	0.075
Log FIRI	0.128	0.237		
Log Fstl1	0.339	0.001	4.355	0.040

BMI: body mass index, BP: blood pressure, LDL: low density lipoprotein, HDL: high density lipoprotein, eGFR: estimated glomerular filtration rate, hsCRP: high sensitive CRP, FIRI: fasting immune-reactive insulin, dROM: derivatives of reactive oxidative metabolites. Multiple model includes all variables at baseline with p<0.1 by single analysis. Data are presented as means ± SD.

## Discussion

In the present study, we demonstrated for the first time that plasma Fstl1 levels were independently correlated with hsCRP and dROMs in healthy male subjects. Treatment with interferon-γ and interleukin-1β is shown to increase Fstl1 secretion in human primary skeletal muscle cells [19]. Treatment of fibroblast-like synoviocytes with tumor necrosis factor-α or interleukin-1β also increases Fstl1 expression [20]. In experimental mouse models, circulating levels of Fstl1 are increased by myocardial ischemic injury and hypertrophy, which are associated with enhanced inflammatory response and oxidative stress [7,8]. Furthermore, increased expression of Fstl1 is observed in cardiomyocytes and endothelial cells in human systolic heart failure [21]. Clinical studies showed that circulating Fstl1 levels are elevated in association with acute coronary syndrome and chronic heart failure [12,13]. These observations suggest that inflammatory and oxidative stress states lead to induction of Fstl1 expression. Furthermore, Fstl1 is shown to protect against cardiovascular and renal injury, and attenuate inflammatory response in ischemic heart and damaged kidney [5–8,10,11,22]. Considering the anti-inflammatory actions of Fstl1, Fstl1 may be upregulated under conditions of inflammation and oxidative stress, presumably due to the counter-regulatory response to alleviate damage of tissues including heart and kidney.

Accumulating evidence has shown that chronic low grade inflammation is involved in the development of metabolic syndrome and atherosclerosis [23–25]. Elevated hsCRP levels are closely associated with insulin resistance and type 2 diabetes, [15,26]. Circulating hsCRP level is also an independent predictor of myocardial infarction and stroke [27]. Furthermore, plasma hsCRP level is a stronger predictor of cardiovascular events compared with LDL cholesterol in apparently healthy women [28]. Our data indicates the strong association between plasma Fstl1 and hsCRP levels in healthy subjects. Thus, circulating levels of Fstl1 could be a useful biomarker of metabolic dysfunction and cardiovascular disease.

Reactive oxygen species (ROS) induces oxidative stress and associates with various pathological conditions including cardiovascular diseases [29,30]. It has been shown that dROMs can be a useful marker for assessment of ROS [18,31]. Elevation of dROMs associates with the presence of coronary artery disease and predicts future cardiovascular events [14]. The higher levels of dROMs are observed in the patients with chronic kidney disease and atrial fibrillation [16,32]. In the present study, plasma Fstl1 concentration was independently correlated with dROMs. These results indicate that Fstl1 may be an indicator for various oxidative stress-related disorders.

It has been controversial whether Fstl1 is pro-inflammatory or anti-inflammatory. Fstl1 is reported to ameliorate joint inflammation in a mouse model of arthritis induced by anti-type II collagen antibody and lipopolysaccharide [33]. Fstl1 suppresses expression of pro-inflammatory genes in a model of heart allograft tolerance [33,34]. Fstl1 negatively regulates expression of inflammatory mediators in the kidney after cisplatin treatment or subtotal nephrectomy [11,22]. Fstl1 also reduces expression of inflammatory cytokines in the heart following ischemia-reperfusion [8]. In contrast, Fstl1 promotes collagen-induced arthritis by enhancing expression of pro-inflammatory cytokines [35–37]. These discrepancies may be explained by the differences in the experimental conditions and Fstl1 target organs.

In conclusion, our study indicates that Fstl1 represents a novel biomarker for inflammatory and oxidative stress responses and that measurement of circulating Fstl1 concentrations could be valuable for evaluation of pathological conditions that are linked with chronic inflammation and oxidative stress.
